# Visualization of coronary arteries in paediatric patients using whole-heart coronary magnetic resonance angiography: comparison of image-navigation and the standard approach for respiratory motion compensation

**DOI:** 10.1186/s12968-019-0525-8

**Published:** 2019-02-25

**Authors:** Mari Nieves Velasco Forte, Israel Valverde, Nanda Prabhu, Teresa Correia, Srinivas Ananth Narayan, Aaron Bell, Sujeev Mathur, Reza Razavi, Tarique Hussain, Kuberan Pushparajah, Markus Henningsson

**Affiliations:** 1 Division of Biomedical Engineering and Imaging Sciences, King’s College London, London, UK; 2 Department of Congenital Heart Disease, Evelina London Children’s Hospital, Guy’s and St Thomas NHS Foundation Trust, London, UK; 3 Cardiovascular Pathology Unit, Institute of Biomedicine of Seville, IBIS, Virgen del Rocio University Hospital/CSIC/University of Seville, Seville, Spain; 4 Department of Pediatrics, University of Texas Southwestern Medical Center, 1935 Medical District Drive, Dallas, USA; 5 Division of Cardiovascular Medicine, Department of Medical and Health Sciences, Linköping University, Linköping, Sweden

**Keywords:** Coronary magnetic resonance angiography, Respiratory motion compensation, Coronary artery disease, Image-based navigation

## Abstract

**Aims:**

To investigate the use of respiratory motion compensation using image-based navigation (iNAV) with constant respiratory efficiency using single end-expiratory thresholding (CRUISE) for coronary magnetic resonance angiography (CMRA), and compare it to the conventional diaphragmatic navigator (dNAV) in paediatric patients with congenital or suspected heart disease.

**Methods:**

iNAV allowed direct tracking of the respiratory heart motion and was generated using balanced steady state free precession startup echoes. Respiratory gating was achieved using CRUISE with a fixed 50% efficiency. Whole-heart CMRA was acquired with 1.3 mm isotropic resolution. For comparison, CMRA with identical imaging parameters were acquired using dNAV. Scan time, visualization of coronary artery origins and mid-course, imaging quality and sharpness was compared between the two sequences.

**Results:**

Forty patients (13 females; median weight: 44 kg; median age: 12.6, range: 3 months–17 years) were enrolled. 25 scans were performed in awake patients. A contrast agent was used in 22 patients. The scan time was significantly reduced using iNAV for awake patients (iNAV 7:48 ± 1:26 vs dNAV 9:48 ± 3:11, *P* = 0.01) but not for patients under general anaesthesia (iNAV = 6:55 ± 1:50 versus dNAV = 6:32 ± 2:16; *P* = 0.32). In 98% of the cases, iNAV image quality had an equal or higher score than dNAV. The visual score analysis showed a clear difference, favouring iNAV (*P* = 0.002). The right coronary artery and the left anterior descending vessel sharpness was significantly improved (iNAV: 56.8% ± 10.1% vs dNAV: 53.7% ± 9.9%, *P* < 0.002 and iNAV: 55.8% ± 8.6% vs dNAV: 53% ± 9.2%, *P* = 0.001, respectively).

**Conclusion:**

iNAV allows for a higher success-rate and clearer depiction of the mid-course of coronary arteries in paediatric patients. Its acquisition time is shorter in awake patients and image quality score is equal or superior to the conventional method in most cases.

**Electronic supplementary material:**

The online version of this article (10.1186/s12968-019-0525-8) contains supplementary material, which is available to authorized users.

## Background

Whole-heart coronary magnetic resonance angiography (CMRA) is commonly used in paediatric patients, especially in those with congenital heart disease (CHD) to assess cardiac morphology and structural disease [1]. This technique allows for volumetric data acquisition with adequate signal-to-noise ratio and spatial resolution that provides crucial diagnostic information for clinical assessment in these patients [2]. However, the extensive scan time of CMRA requires acquisition during free-breathing and respiratory motion compensation techniques to mitigate motion artifacts [3, 4]. The most common approach involves the use of an interleaved one-dimensional respiratory ‘navigator’ acquisition positioned on the right hemi-diaphragm. The diaphragmatic navigator (dNAV) can be used to gate the CMRA scan by discarding and re-acquiring CMRA data, which falls outside a pre-defined end-expiratory gating window [5]. The dNAV can also be used to correct for the respiratory motion by employing a linear tracking factor between the respiratory motion of the diaphragm and that of the heart, often assumed to be 0.6 [6].

Recently, advanced CMRA motion compensation strategies have been proposed to improve image quality [7], reduce scan time [8], or both [9]. These primarily involve measuring the respiratory motion directly on the heart, rather than the diaphragm. Direct motion measurements have been achieved using self-navigation [10] where the navigator is extracted from the CMRA data or image-based navigation (iNAV) where two-dimensional or three-dimensional real-time images are interleaved with the CMRA scan [11–14]. Similar to dNAV, gating can be combined with iNAV to mitigate respiratory motion artifacts and improve image quality at the expense of scan time. Image-based navigation was demonstrated to be superior to dNAV in adult patients with congenital heart disease, in a recent study [9]. However, the iNAV technique relied on an external bellows signal for respiratory gating. More recently, respiratory gating has been implemented for the iNAV utilizing Constant Respiratory Using Single End-expiratory Thresholding (CRUISE), allowing gating with a fixed efficiency without relying on external signal [15]. The main advantages of CRUISE compared to external respiratory bellows gating are the simplified and fully inline scan setup, predictable scan time, and avoidance of potential hysteresis and poor correlation between bellows and heart motion. The aim of this work was to investigate the use of iNAV CRUISE for CMRA motion compensation and compare it to the conventional dNAV in paediatric patients with CHD or suspected heart disease.

## Materials and methods

### Patient selection

The study was approved by the National Research Ethics Service (IRB: 10/H0802/65) and all participants provided written informed consent. Between March 2016 and October 2016 consecutive pediatric patients with congenital heart disease referred to Evelina Children’s Hospital, London, UK for cardiovascular magnetic resonance (CMR) were considered for inclusion. Patients with haemodynamic instability or referred from intensive care unit were not included in the study in order to avoid any increase in general anaesthetic (GA) time.

### CMR protocol

All experiments were performed on a 1.5 T clinical scanner (Achieva, Philips Healthcare, Best, The Netherlands) using a 5-channel cardiac coil. The CMR protocol was modified based on the specific clinical indications, but typically included multi-slice cine, phase contrast flow, black blood vessel wall imaging, and dynamic contrast enhanced CMRA. Dotarem® (Gadoterate meglumine; concentration: 0.5 mmol/ml of gadolinium; dose: 0.2 mmol/kg) or Gadovist® (gadobutrol, concentration: 1.0 mmol/ml; dose: 0.1 mmol/kg) contrast agents were used as required depending on the indication for CMR scan. If dynamic contrast enhanced CMRA was performed, it was followed immediately by the CMRA scans using iNAV and dNAV in a randomised order. General anaesthesia was utilized when clinically indicated as per our institutional protocol.

The CMRA used balanced steady-state free precession (bSSFP) readout with the following imaging parameters: field-of-view = 250–300 × 250–300 × 60-100 mm^3^, acquired resolution = 1.3 × 1.3 × 1.3 mm^3^, repetition time/echo time = 3.9/1.95 ms, flip angle = 70°, and parallel imaging acceleration factor = 2 (in-plane phase encoding direction). Vector-cardiography (VCG) triggering was used to minimize cardiac motion, with subject-specific trigger delays and acquisition windows using the longest rest period at a single phase of the cardiac cycle. To improve CMRA contrast, T2 prep (echo time = 35 ms) and fat suppression pre-pulses were used. Using these imaging parameters, and assuming a heart-rate of 80 bpm and 100 ms acquisition window, the nominal scan time was 3 min and 20 s. The CMRA scan with dNAV motion compensation used a tracking factor of 0.6 and gating window according to patient’s weight (3 mm if less than 20 kg, 5 mm if between 20 kg and 40 kg, and 7 mm if more than 40 kg) for all patients. The CMRA scan with iNAV motion compensation is described in the following section.

### Image-navigated CMRA motion compensation

The acquisition of iNAV was performed by adding phase encoding gradients to the 10 startup echoes of the bSSFP sequence [11]. A region-of-interest encompassing the whole heart was tracked in foot-head (FH) and left-right (LR) direction, and selected using the local shim geometry. The iNAV reference was defined as the first acquired navigator to which all subsequent iNAVs were registered using normalised cross-correlation. The 2D translational correction was applied to the CMRA k-space raw data by modulating its phase. Respiratory gating was implemented using CRUISE [15]. In brief, this approach acquires twice as much data as needed to fill CMRA k-space (resulting in exactly 50% gating efficiency) and only the half acquired at the most end-expiratory was used to reconstruct the gated image. Both iNAV correction and gating was performed in real-time on the scanner, and no post-processing was required.

### Image analysis

All CMRA images were reformatted using dedicated software [16] to visualize the right coronary artery (RCA), left main, left anterior descending artery (LAD), and left circumflex artery (LCX). Images were objectively and subjectively analysed in terms of [1] image quality [2], sharpness of the vessel [3] identification of the coronary artery origins [4], length of coronary arteries (quantitative and qualitative assessment).

A visual score was used, based on a previous CMRA patient study [17], to qualitatively assess overall CMRA image quality using the following scale: 1 – coronary artery poorly visualized, 2 – coronary artery visible but with marked blurring, 3 – coronary artery visible with moderate blurring, 4 – coronary artery visible with mild blurring, and 5 – coronary artery visible with sharp edges. Analysis of the visual score and course of the coronary arteries was performed using iNAV and dNAV 3D datasets for all patients by three independent, blinded observers (MNV, IV and TH). All observers had 5 years or more of experience in CMR imaging analysis. Intra- and inter-observer agreement was calculated. Absolute agreement for image quality was noted when both observers gave exactly the same score and relative agreement was defined by scores differing by no more than one point. Inter-observer agreement was reported as mean comparisons across all 3 observer combinations (ie MNV vs IV; IV vs TH and MNV vs TH).

The vessel sharpness was calculated on the first 4 cm of all coronary arteries, as a percentage where 0% equals no edge and 100% a step edge, using dedicated software [16] by an expert with 9 years of experience in CMRA (MH). Vessel sharpness was performed by a second expert (TC) for both techniques on 10 random patients, resulting in a total of 60 analysed vessels. The readers were blinded to the motion correction method used when performing the vessel sharpness analysis. A subgroup analysis was performed on the coronary vessel sharpness measurements, where patients scanned during general anaesthesia were analysed separately from awake patients. Furthermore, another subgroup analysis was performed, separating scans performed using contrast agents from scans without.

**Fig. 1:**
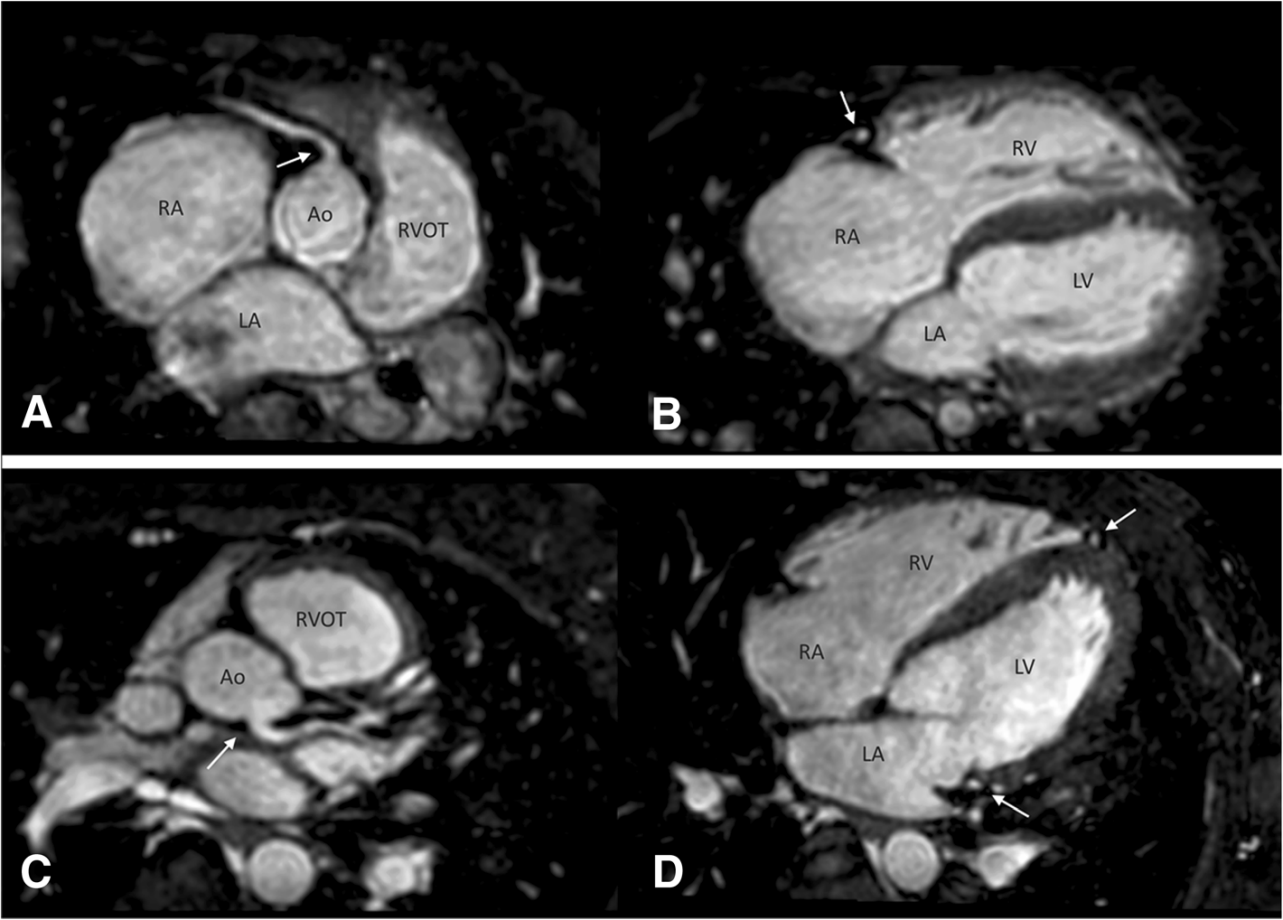
Analysis of origin and mid-course visualization of right coronary artery (RCA; superior image) and left (inferior) coronary arteries. **a** Origin of the RCA (arrow) and proximal course. **b** Mid-course visualization of the RCA (arrow) in the same patient. **c** Origin (arrow) and proximal course of the LCA. **d** Mid-course of the left anterior descending (LAD) and left circumflex (LCX) (arrows) in the same patient

For each patient, we assessed whether the origin, proximal and mid-course of the coronary arteries were visible, on a multiplanar reformat. Proximal visualization was considered satisfactory when the origins of both coronary arteries were observed. Mid-course depiction was described for each coronary artery as represented in Fig. 1: for the RCA and LCX, visualization was considered successful when their course was visualised up to the mid-point of the atrioventrictular (AV) groove using the AV valve as a reference point; for the LAD, its course was followed along the ventricular septum up to same point as the LCX. Also the length of each coronary artery was quantitatively measured using dedicated software [3].

### Statistical analysis

Statistical analysis was performed using MATLAB (The Mathworks Inc., Natick, Massachusetts, USA) statistics toolbox and SPSS (v 20.0, International Business Machines, Armonk, New York, USA). For the continuous variables vessel sharpness and scan time, a two-tailed t-test was performed to evaluate statistical significance. Continuous variables are presented as mean ± standard deviation unless specified otherwise. Proximal and distal visualization was compared between iNAV and dNAV datasets as a proportion of success rate among all visualizations performed by the 2 observers. For the categorical variable (visual score) a Wilcoxon signed rank test was performed to evaluate statistical significance. Categorical variables are presented as median, 75th percentile, 25th percentile. A *P* value less than 0.05 was considered statistically significant. A Holm-Bonferroni correction was performed where multiple comparisons were used.

## Results

**Fig. 2:**
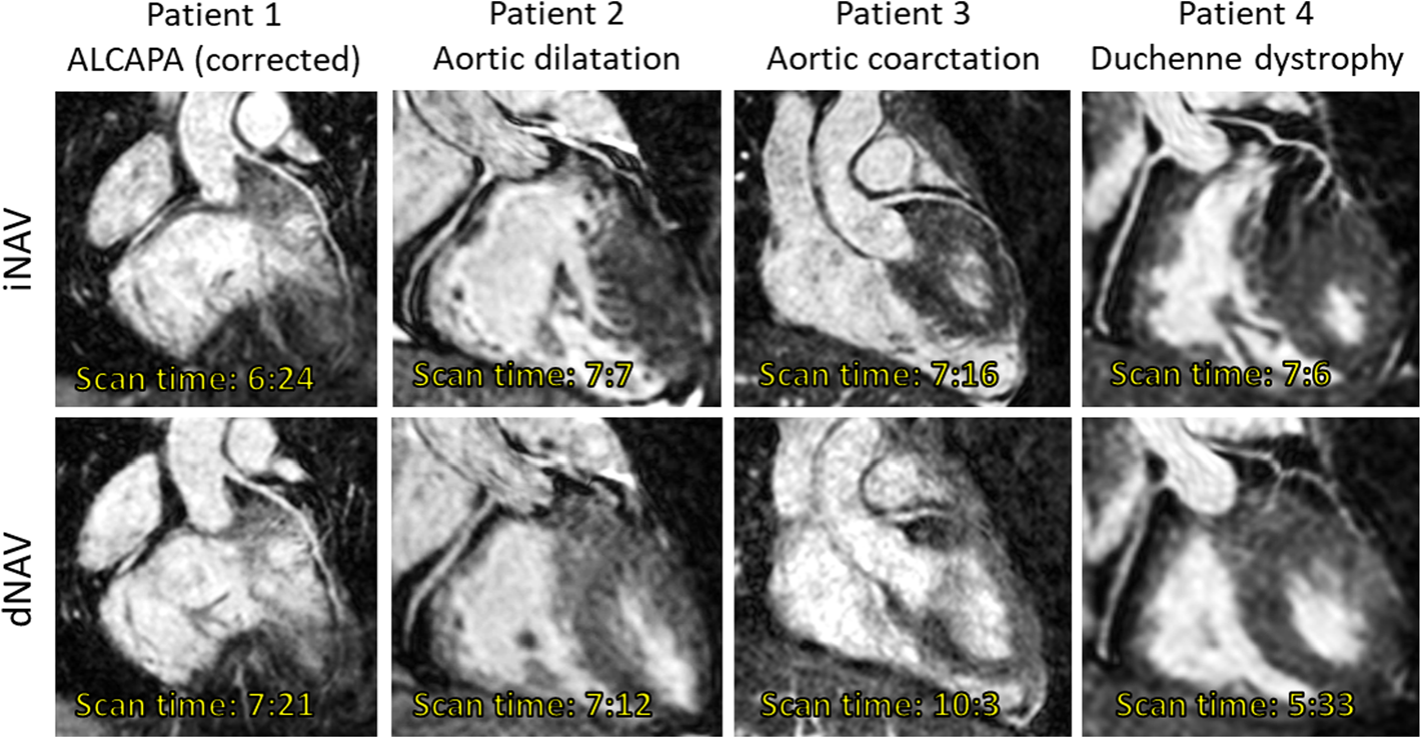
Whole-heart coronary magnetic resonance angiography (CMRA) in a 15-year old male with a body mass index (BMI) of 22 (Patient 1), 16-year old male with a BMI of 22 (patient 2), 14-year old female with a BMI of 19 (Patient 3), and 16-year old male with a BMI of 21 (Patient 4). CMRA acquisition was performed using image-based respiratory navigation (iNAV, top row) and conventional diaphragmatic one-dimensional navigation (dNAV, bottom row)

A total of 40 patients (27 males; median weight: 44 kg; range: 4–80; median age: 12.6 years, range: 3 months–17 years) were enrolled. There was no significant difference in age according to the sex of the patients (females: mean 11.4 years, range: 2.8 months – 15.6 years; males: 10.3 years, range: 3.8 months – 17 years, *P* > 0.5). Diagnosis and demographic features are summarised in Table 1. An extended explanation of diagnostic characteristics is provided in Additional file 1. Twenty-five scans were performed in awake patients and 15 under general anesthesia. A contrast agent was used in 22 cases, 6 using Gadovist and 16 using Dotarem. All patients were in sinus rhythm and median heart rate during scan acquisition was 75 beats per minute (range: 59–110). Representative images from 4 patients are shown in Fig. 2.

**Table 1:** Demographic features and diagnosis for patients according to gating window used during dNAV scanning

Gating window (dNAV)	Weight (kg)	BSA (m2)	HR (bpm)	Age (years)	Sex	Diagnosis	N
3 mm	12 ± 5.2	0.53 ± 0.18	83 ± 15	3.1 ± 2.6	2 females	- 1 DILV, TGA, CoA - 6 CHD with GV involvement (including TOF, DORV &TGA) - 1 dextrocardia, ccTGA, PS - 1 BAV, AoCo - 1 VSD, LV non-compaction, Brugada	10
5 mm	29 ± 6.7	1.03 ± 0.15	80 ± 10	9.2 ± 1.7	2 females	- 1 TOF - 1 DORV - 3 GV disease	5
7 mm	56.6 ± 11.3	1.6 ± 0.22	76 ± 12	14 ± 2.3	9 females	- 4 Arrhythmias/ cardiomyopathy - 15 CHD with GV involvement ± VA valve disease (including TOF) - 2 AV valve disease - 2 HLHS - 2 ALCAPA syndrome	25

*ALCAPA* Anomalous Left Coronary Artery from the Pulmonary Artery, *CoA* Coarctation of the aorta, *AV* atrioventricular, *BAV* bicuspid aortic valve, *CHD* congenital heart disease, *DILV* double inlet left ventricle, *DORV* double outlet right ventricle, *GV* great vessels, *HLHS* hypoplastic left heart syndrome, *PS* pulmonary stenosis, *LV* left ventricle, *TGA* transposition of the great arteries, *ccTGA* congenitally corrected transposition of the great arteries, *TOF* tetralogy of Fallot, *VA* ventriculoarterial, *VSD* ventricular septal defect

### Scan time

**Fig. 3:**
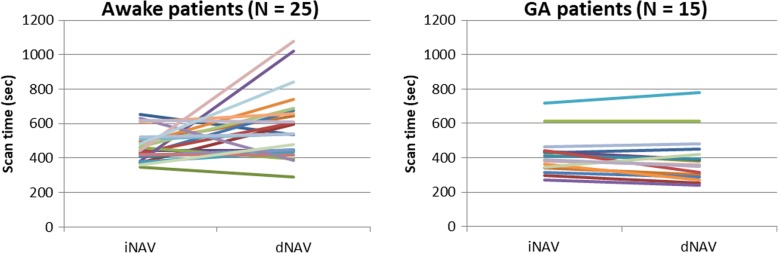
Scan times for awake patients (left), and patients under general anaesthesia (right), using iNAV and dNAV

The scan time was significantly shorter using iNAV [min:sec] (6:59 ± 1:23) compared to conventional dNAV (9:17 ± 2:34, *P* < 0.05). Subgroup evaluation between awake and patients under GA demonstrated that shorter acquisition time was significantly reduced only for awake patients (*n* = 25) (iNAV 7:48 ± 1:26 vs dNAV 9:48 ± 3:11, *P* = 0.01). However, there were no significant differences in scan time for patients under general anesthesis (iNAV = 6:55 ± 1:50 versus dNAV = 6:32 ± 2:16; *P* = 0.32). The scan time for awake and general anesthesia patients are shown in Fig. 3.

### Image quality

**Fig. 4:**
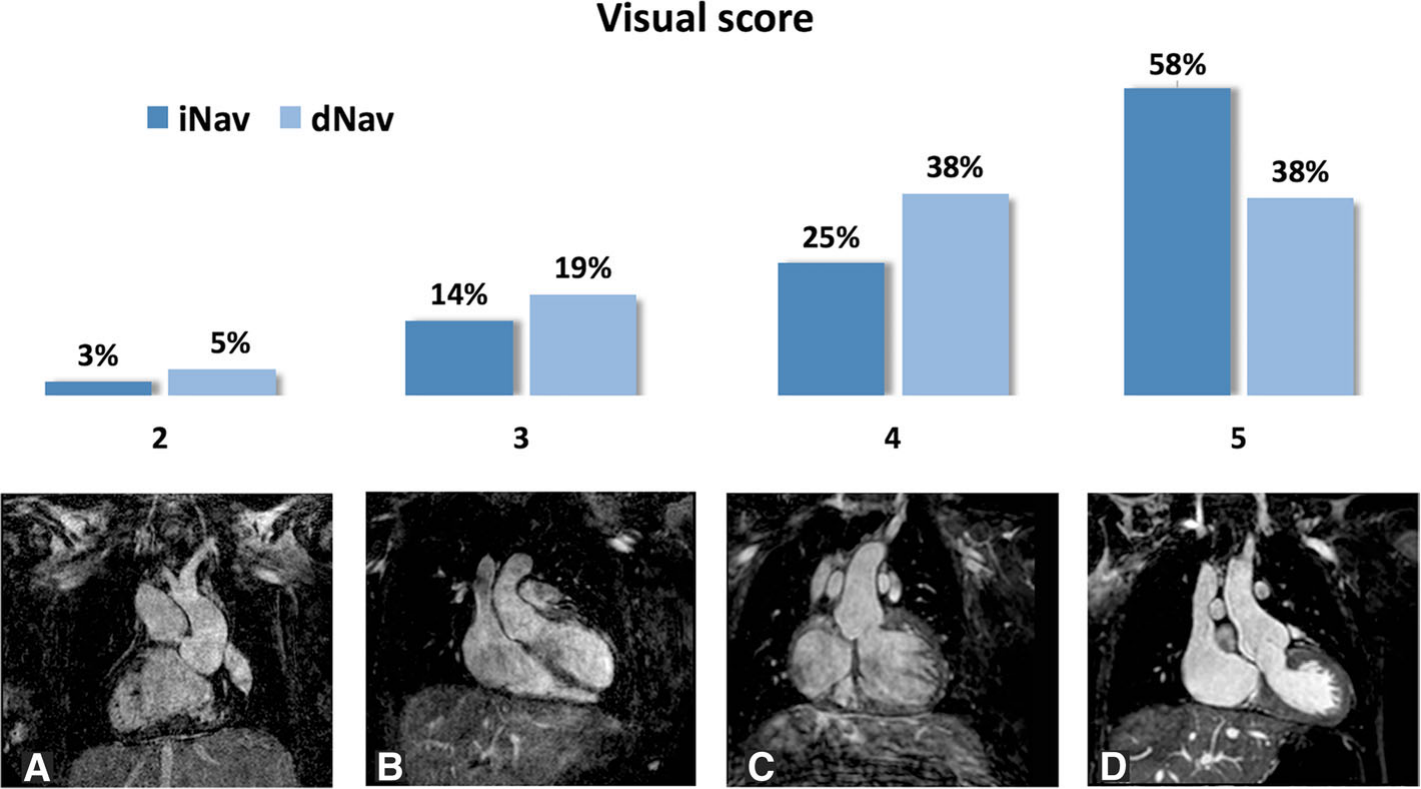
Visual score for images acquired with iNAV and dNAV. Percentage of cases scored from 1 to 5 for both modalities is shown at the top of the image. Figures **a**-**d** represent scores from 2 to 5, respectively. No images received a score of 1

In 39 out of 40 cases (98%), iNAV image quality had a similar or higher score than images acquired with dNAV. Intra and inter-observer image quality absolute agreement (where both observed scores are exactly the same) was excellent at 92.5% (95% confidence interval 86.7, 98.3) and 81.7% (95% confidence interval 76.8, 86.6) respectively. Relative agreement (where observed scores differed by no more than one point) was 100% for intra-observer measurements and 100% for inter-observer measurements. The analysis of the visual score with Wilcoxon test showed a statistically significant difference in favour of iNAV versus dNAV (*P* = 0.002). The results of the visual score for all patients are summarized in Fig. 4.

### Vessel sharpness

**Fig. 5:**
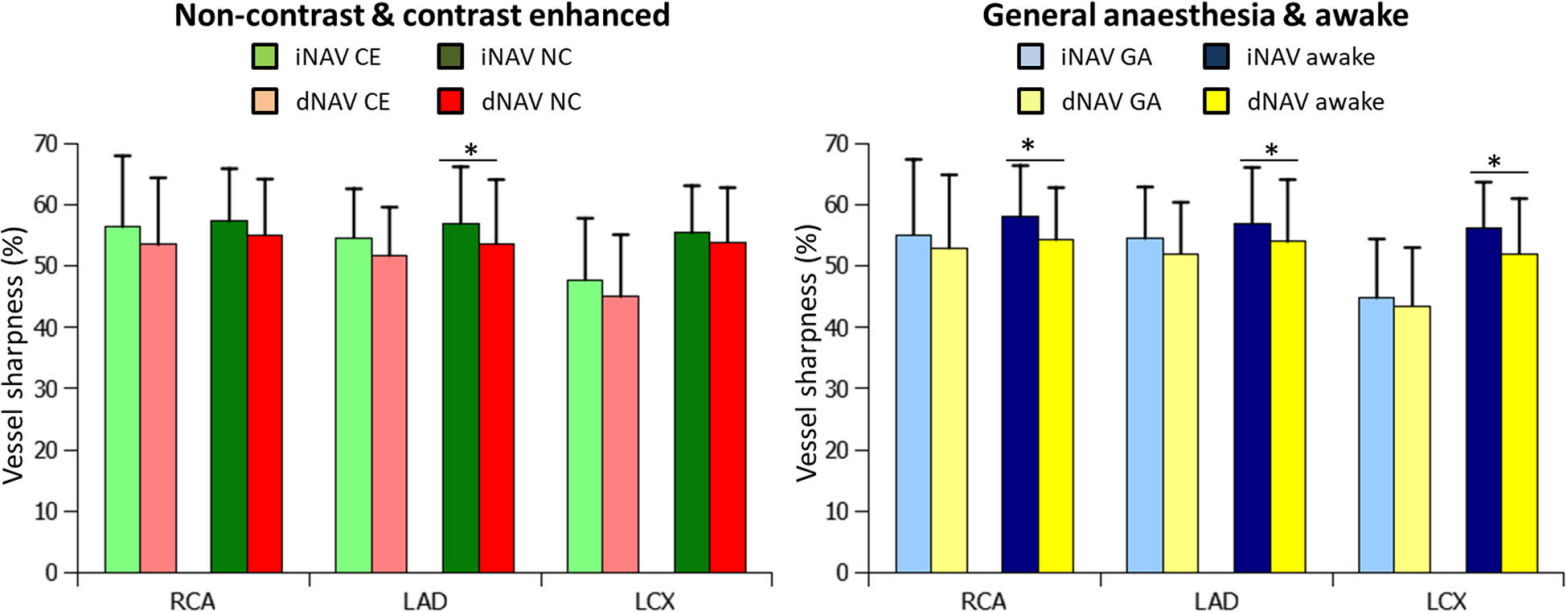
Coronary artery vessel sharpness, analysed separately in patient with (*n* = 22) and without (*n* = 18) contrast agent (left graph) for iNAV and dNAV, and in patients scanned under general anaesthesia (*n* = 15) and awake (*n* = 25) patients (right graph). * denotes a statistically significant difference

In the combined analysis of all 40 patients, the sharpness of the vessels was significantly higher with iNAV for the RCA and the LAD (iNAV: 56.8 ± 10.1% vs dNAV: 53.7 ± 9.9%, *P* < 0.002, and iNAV: 55.8 ± 8.6% vs dNAV: 53 ± 9.2%, *P* = 0.001, respectively). However, there was no significant difference for the LCX (iNAV: 52.2% ± 9.8% vs dNAV: 49.1% ± 10.1%, *P* = 0.18). The subgroup analysis of patients scanned with contrast agents (*n* = 22) and those without (*n* = 18) yielded a statistically significant difference for the LAD in patients without contrast agents, where the iNAV performed better than the dNAV (iNAV: 56.9% ± 9.3% vs dNAV: 53.6% ± 10.5%, *P* = 0.01). The subgroup analysis of patients scanned under general anaesthesia (*n* = 15) and those awake (*n* = 25) resulted in a statistically significant difference for the RCA (iNAV: 58% ± 8.4% vs dNAV: 54.3% ± 8.5%, *P* = 0,005), LAD (iNAV: 57% ± 9.1% vs dNAV: 54% ± 10.1%, *P* = 0,01) and LCX (iNAV: 56.3% ± 7.4% vs dNAV: 52% ± 9%, *P* = 0,01) in awake patients. The subgroup analysis of the vessel sharpness measurements is shown in Fig. 5. The inter-observer agreement for the coronary vessel sharpness measurements was good, and similar for both iNAV and dNAV, with a small bias of 0.3% for iNAV and 0.5% for dNAV. The 95% confidence interval was 6.9% to − 6.4% for iNAV and 7.4% to − 6.5% for dNAV.

### Identification of coronary artery origins and visualized coronary artery length

Although the coronary artery origins were depicted in all patients independently of the type of motion correction strategy that was utilized, quantitative analysis of the length of the coronary arteries using iNAV demonstrated a significant improvement in the length of the coronary arteries visualized for the RCA (iNAV: 6.6 mm ± 0.49 mm vs dNAV: 6.0 mm ± 0.4 mm, *P* < 0.05) and the LAD (iNAV: 7.3 mm ± 0.6 mm vs dNAV: 6.6 mm ± 0.6 mm, *P* < 0.01) with no significant difference in the case of the LCX (iNAV: 5.2 mm ± 0.5 mm vs dNAV: 4.9 mm ± 0.4 mm, *P* = 0.27).

In agreement with numerical analysis, mid-course assessment from CMR clinicians showed improved results when using iNAV. The mid-course of the coronary arteries was not visualized in any of the coronary arteries in 4% using dNAV (vs 0% using iNAV) and was visualized in only one or two branches in 34% (vs 16% using iNAV). Subsequently, a complete visualization in all 3 coronary arteries was possible using iNAV in 84% of cases, and 63% using dNAV. An individual analysis for each coronary artery revealed a significant difference for each one independently when comparing mid-course visualization with iNAV and dNAV. The mid-course of the RCA was visualized in 95% of the cases using iNAV and 85% with dNAV (*P* < 0.05). This proportion was 89% (iNAV) vs 76% (dNAV) for the LAD (*P* < 0.05) and 94% vs 70% for LCx (*P* < 0.001).

## Discussion

In this study, we have evaluated a new method for respiratory motion compensation in a heterogeneous population of paediatric patients demonstrating improvements in image quality compared to the conventional motion compensation technique. Furthermore, we observed a significant reduction in CMRA scan time in awake patients using the proposed motion compensation technique. The reduced scan time using iNAV compared to dNAV is due to the CRUISE gating strategy which assumes a constant 50% efficiency, regardless of patient breathing pattern. Conversely, dNAV uses a predefined constant gating window (based on the patient weight), resulting in a variable efficiency depending on the breathing pattern. This is particularly pertinent in non-anaesthetised patients where the gating efficiency may change during the scan, potentially leading to excessive scan times. In this cohort of patients there was a significant reduction in scan time using iNAV with CRUISE gating, compared to gated dNAV, of approximately 25%. Importantly, for the non-anaesthetised patients the standard deviation of the scan time using iNAV CRUISE was less than half of the gated dNAV scans, suggesting the scan time is much more predictable with this approach. A typical total examination time in these patients is approximately an hour, with an estimated 5–10 min allocated for whole-heart CMRA. Although the average dNAV scan time in awake patients is within the upper bound of this range, there is a high variability with a standard deviation of over 3 min. With the dNAV technique we recorded scan times of 18 min which would increase the total examination time by approximately 15%. For general anesthesia patients there was no significant difference in scan time between the two techniques. This can be explained by the stable breathing pattern of patients under general anesthesia leading to a constant gating efficiency even using a narrow gating window, which is the case for dNAV. Similarly, the scan time standard deviation was approximately the same using iNAV CRUISE and gated dNAV in this cohort.

As revealed in the subgroup analysis of vessel sharpness scores, iNAV appears to perform particularly well relative to dNAV in awake patients where statistically significant improvements were found for the RCA, LAD and LCX. However, for the entire patient cohort, including awake patients and those under general anesthesia, statistically significant coronary sharpness improvements were only observed for the RCA and LAD using iNAV. This highlights the important role of adequate respiratory motion correction in awake patients, where irregular breathing, unpredictable image quality and scan times are often observed using the conventional technique.

The use of iNAV in adult patients with CHD has been previously reported [9]. However, the previously proposed iNAV technique was limited to respiratory gating using an external respiratory bellows’ measurement. In contrast, the proposed iNAV employs inline gating using the iNAV measurements, thereby simplifying scan setup and improving gating performance. In a recent study by Monney et al. [18] respiratory self-gating was used in 105 CHD patients with successful visualisation of the origin and proximal course of RCA, LAD and LCX in 93, 87 and 98%, respectively. In this study, both iNAV and dNAV achieved 100% success rate for visualization of origin and proximal course for all coronary arteries. In our study, the mid-course was visualized in 95, 89 and 94% for the RCA, LAD and LCX respectively when using iNAV. These results showed a significant difference compared to dNAV, in which mid-course visualization was only achieved in 85, 76 and 70% of the visualizations among cases, respectively.

The coronary vessel sharpness was found to be improved using iNAV compared to dNAV. This is consistent with the results reached in subjective scoring, where most of the cases had better or equal image quality in the analysis of the images acquired with iNAV in relation to dNAV.

Apart from the quantifiable improvements in image quality and scan time, iNAV also provided improvements in CMRA ease-of-use as no dedicated scan planning is required for the navigator. The tracked region of interest (ROI) is defined as the local shim geometry which is typically the same for all scans throughout the CMR examination and planned to encompass the heart and great arteries. Due to the real-time iNAV reconstruction and motion feedback, the scan can be stopped and ROI re-defined if the motion tracking is deemed inadequate. However, during the study re-planning of the iNAV ROI was not necessary in any of the patients.

Although the proposed iNAV technique improves the quality of the images for visualization of coronary arteries and extends its depiction to their mid-course, the distal coronary anatomy is still difficult to delineate in CMR. Multi Detector Computer Tomography (MDCT) remains a better tool for this purpose especially in children, owing to the higher spatial resolution, variability in the heart rate and the possibility of reconstruction over multiple cardiac cycles when retrospective acquisition is applied [1, 19]. However, despite offering high quality images, coronary MDCT angiography involves radiation and requires iodinated contrast in all patients [20]. Efforts are therefore being made in order to improve coronary anatomy and wall motion characterization with CMR, particularly in children with CHD [2], as they constitute a vulnerable population given the need for repeated diagnostic and interventional procedures, where the definition of the proximal coronary anatomy is vital [21, 22]. In this regard, we have previously shown the accuracy of the 3d whole-heart technique in imaging the proximal course of the coronary arteries in these cases [23–25]. We have also previously shown the applicability in the morphological diagnosis of these patients [26, 27]. Given the known high accuracy of the technique, in this study, we chose to demonstrate an improvement in image quality as a surrogate for improving accuracy further. Indeed, to show an improvement in accuracy would require a much larger study for adequate statistical power.

There is currently a trend within the research community towards continuously acquired multi-phase CMRA, which allows retrospective selection of the optimal phase to visualize the coronary arteries. Typically, continuous CMRA acquisitions rely on a 1D self-navigation signal for beat-to-beat motion correction [28–30]. A limitation of iNAV in this context is that the temporal footprint (approximately 40 ms) encroaches on the time available to acquire CMRA data, which may lead to a lower temporal resolution or prolonged scan time. However, there are drawbacks associated with continuous CMRA acquisitions compared to single-phase CMRA. Primarily, signal-to-noise ratio and contrast-to-noise ratio are lower in continuous CMRA due to the repeatedly performed RF-pulses throughout the cardiac cycle [31]. In comparison, single phase CMRA has intrinsically high signal-to-noise ratio due to the T1 recovery between acquisition windows. Although exogenous contrast agents may be used to improve the signal-to-noise ratio in continuous CMRA, there is increased vigilance regarding the use of gadolinium-based agents due to potential risks of long-term retention, while iron-oxide based agents are also associated with potential for adverse events.

Our study has other limitations. Firstly, the patient cohort was limited to a relatively small number and from a single centre. Due to the small sample size no sub-group analysis was performed to assess the influence of factors such as general anaesthesia, contrast agent, age, body-mass index or heart rate on image quality. A larger cohort of patients, from multiple centres would be valuable to confirm our current findings, establish the diagnostic performance and clinical utility of this technique. Second, the size of the gating window used for respiratory motion correction in conventional dNAV 3D bSSFP acquisition varied between patients as was established according to the weight of the child following our protocol in clinical scans. These empirically determined gating windows were used to ensure a reasonably constant gating efficiency of approximately 50%, and subsequently a predictable scan time.

## Conclusion

iNAV offers better quality images for coronary visualization in children with congenital or suspected heart disease. The mid-course of the coronary arteries is more often visualized with this method. The reduction in scan time is an advantage in awake patients, particularly in children, in whom the cooperation to remain still for long periods of time is limited.

## Additional file

Additional file 1: **Table S1.** Diagnostic features and CMR indications for all 40 patients. (DOCX 25 kb)

## Abbreviations

AV: Atrioventrictular
bSSFP: Balanced steady-state free precession
CHD: Congenital heart disease
CMR: Cardiovascular magnetic resonance
CMRA: Coronary magnetic resonance angiography
CRUISE: Constant respiratory efficiency using single end-expiratory thresholding
dNAV: Diaphragmatic navigator
FH: Foot-head
iNAV: Image-based navigation
LAD: Left anterior descending coronary artery
LCX: Left circumflex coronary artery
MDCT: Multi detector computer tomography
RCA: Right coronary artery
RL: Right-left
ROI: Region of interest
VCG: Vector-cardiography
