# Supplementary material for: Visualization of coronary arteries in paediatric patients using whole-heart coronary magnetic resonance angiography: comparison of image-navigation and the standard approach for respiratory motion compensation
Source: J Cardiovasc Magn Reson. 2019 Feb 25;21:13. doi: 10.1186/s12968-019-0525-8 (PMC6388473; doi:10.1186/s12968-019-0525-8)
Supplement: Supplementary file 1 — Table S1. Diagnostic features and CMR indications for all 40 patients. (DOCX 25 kb) [file 12968_2019_525_MOESM1_ESM.docx]

**Additional file 1: Table S1**

| **Patient** | **Age (years)** | **Weight (kg)** | **Diagnostic features** | **Indication for CMR** |
| --- | --- | --- | --- | --- |
| **1** | 13 | 53 | Family history of ARVC  Palpitations | Presence of ARVC criteria in CMR |
| **2** | 7 | 47 | Chest pain  Bicuspid aortic valve  Aortic coarctation | LV functional analysis  LV mass  Presence of scar |
| **3** | 15 | 55 | TGA | Coronary arteries, valvular and ventricular assessment |
| **4** | 15 | 72 | Aortic coarctation  Bicuspid aortic valve – mild AR | LV mass analysis  Valvular assessment |
| **5** | 13 | 42 | Alcapa syncrome, moderate mitral regurgitation | LV function  MR quantification |
| **6** | 0.41 (5 months) | 6.2 | DILV/ TGA/ VSD  Aortic coarctation | Preoperative study  Location of VSD  Ventricular size and function |
| **7** | 13 | 53 | Tricuspid regurgitation  Previous endocarditis | Ventricular function and volume  TR quantification |
| **8** | 6 | 22 | Tetralogy of Fallot | PA branches study  RV function  PR quantification |
| **9** | 14 | 70 | Mild LVH  Athlete | LV mass and function analysis |
| **10** | 15 | 75 | TGA  Moderate PAs stenosis | Assessment of MPA and PA branches |
| **11** | 10 | 34 | Mild ascending aorta dilatation | Ascending aorta assessment for on-going dilatation |
| **12** | 9 | 39 | Aortic coarctation | LV mass and function  Aortic arch study |
| **13** | 12 | 60 | ccTGA | Ventricular function and valvular study |
| **14** | 15 | 76 | Dilated ascending aorta  Pectus carinatum | Ascending aorta assessment for on-going dilatation |
| **15** | 17 | 51 | Bicuspid aortic valve  Dilated ascending aorta  Previous aortic valve endocarditis | LV mass and functional analysis  AR quantification |
| **16** | 2 | 15 | Perimembranous VSD  Brugada syndrome  LV non-compaction | Assessment of compacted/non compacted myocardium  LV function |
| **17** | 13 | 44 | ccTGA,  Left SVC  Subpulmonary stenosis | Ventricular function and valvular study |
| **18** | 16 | 51 | Duchenne muscular dystrophy  Mild LV function impairment | LV functional analysis |
| **19** | 15 | 62 | TGA  PAs stenosis | Assessment of PA branches |
| **20** | 3 | 11 | Tetralogy of Fallot  Severe pulmonary regurgitation | Ventricular function and size  PR quantification |
| **21** | 13 | 60 | Dilated ascending aorta  Connective tissue disorder | Ascending aorta assessment for on-going dilatation |
| **22** | 4 | 14 | Dextrocardia  ccTGA  Multilevel pulm stenosis  Asplenia | Ventricular function and valvular study  Assessment of PA branches |
| **23** | 8 | 17.5 | Bicuspid aortic valve  Aortic Coarctation | LV functional analysis and mass quantification |
| **24** | 14 | 60 | Alcapa syndrome | LV functional analysis  MR quantification |
| **25** | 1 | 10 | DORV | Preoperative assessment  Ventricular volumes  VSD location and relation to great arteries |
| **26** | 15 | 44 | PDA | Ventricular volumes  Qp: Qs quantification |
| **27** | 8 | 42 | Supravalvar aortic stenosis  Aortic arch hypoplasia | LV function and valvular study |
| **28** | 0.24 (2 months) | 4.3 | TOF  Right aortic arch  PDA | Preoperative assessment  RV volume  MPA and PA branches assessment |
| **29** | 10 | 28 | DORV | Ventricular and valvular assessment |
| **30** | 9 | 22 | TGA - ST changes during exercise | Coronary artery assessment  LV function |
| **31** | 5 | 19 | TGA  Mild aortic root dilatation | Coronary artery assessment  Ascending aorta assessment for on-going dilatation |
| **32** | 14 | 58 | Left atrial isomerism  AVSD  Dextrocardia  Moderate AVVR | Assessment of Fontan circulation |
| **33** | 15 | 41 | TOF  Pulmonary regurgitation  RV dilatation | Ventricular function and size  PR quantification |
| **34** | 15 | 44 | HLHS | Assessment of Fontan circulation |
| **35** | 12 | 62 | TOF  Pulmonary regurgitation  RV dilatation | Ventricular function and size  PR quantification |
| **36** | 12 | 50 | TOF  Pulmonary regurgitation  RV dilatation | Ventricular function and size  PR quantification |
| **37** | 0.32 (3 months) | 5 | Tetralogy of Fallot  Critical pulmonary stenosis  Hypoplastic pulmonary arteries | Ventricular function and size  MPA and PA branches assessment |
| **38** | 6 | 18 | TOF  Pulmonary regurgitation  RV dilatation | Ventricular function and size  PR quantification |
| **39** | 14 | 80 | Mild ascending aorta dilatation  Bicuspid aortic valve | Ascending aorta assessment for on-going dilatation |
| **40** | 17 | 63 | Supraventricular tachycardia  Tachymyopathy | LV functional analysis |
